# High-Resolution Neural Network for Driver Visual Attention Prediction

**DOI:** 10.3390/s20072030

**Published:** 2020-04-04

**Authors:** Byeongkeun Kang, Yeejin Lee

**Affiliations:** 1 Department of Electronic and IT Media Engineering, Seoul National University of Science and Technology, Seoul 139-743, Korea; byeongkeun.kang@seoultech.ac.kr; 2 Department of Electrical and Information Engineering, Seoul National University of Science and Technology, Seoul 139-743, Korea

**Keywords:** saliency estimation, visual attention estimation, driver perception modeling, intelligent vehicle system, convolutional neural networks

## Abstract

Driving is a task that puts heavy demands on visual information, thereby the human visual system plays a critical role in making proper decisions for safe driving. Understanding a driver’s visual attention and relevant behavior information is a challenging but essential task in advanced driver-assistance systems (ADAS) and efficient autonomous vehicles (AV). Specifically, robust prediction of a driver’s attention from images could be a crucial key to assist intelligent vehicle systems where a self-driving car is required to move safely interacting with the surrounding environment. Thus, in this paper, we investigate a human driver’s visual behavior in terms of computer vision to estimate the driver’s attention locations in images. First, we show that feature representations at high resolution improves visual attention prediction accuracy and localization performance when being fused with features at low-resolution. To demonstrate this, we employ a deep convolutional neural network framework that learns and extracts feature representations at multiple resolutions. In particular, the network maintains the feature representation with the highest resolution at the original image resolution. Second, attention prediction tends to be biased toward centers of images when neural networks are trained using typical visual attention datasets. To avoid overfitting to the center-biased solution, the network is trained using diverse regions of images. Finally, the experimental results verify that our proposed framework improves the prediction accuracy of a driver’s attention locations.

## 1. Introduction

As driving is a task that demands heavy-duty visual information processing, human vision plays the most important role in making suitable decisions for vehicle control. As such, comprehensively understanding a driver’s visual behavior is an essential component for the guidance of safe driving in advanced driver-assistance systems (ADAS) and autonomous intelligent vehicle systems.

Human drivers continuously aware of situations when making decisions. In particular, drivers give attention to surrounding objects (e.g., vehicles, pedestrians, traffic signs, etc.) based on scene-specific, task-related, and object-level cues [1]. Similarly, it has been shown in the literature that a driver’s actions are strongly linked to where the driver’s visual attention is focused [2,3]. Thus, the attention required by a driver is a crucial concern of safe driving and motivates this work. A driver’s visual attention behavior is mainly accountable using biological evidence. The direction of a driver’s visual attention involves processing of information from regions of interest as well as processing of relevant surrounding information [4,5,6]. This visual processing is performed by two different types of vision, namely central and peripheral. Central vision (also known as “foveal vision”) has the highest acuity, where scene details are perceived in sharp. An example of central vision function in driving is seeing pedestrians in focus. On the other hand, peripheral vision (also known as “indirect vision”) allows us to view around focus without turning heads or moving eyes. An example of peripheral vision function is noticing pedestrians approaching while the driver’s attention is focused on the road [7]. Combining two visions, a driver determines where he/she allocates his/her subsequent attention.

Extensive research has been explored to understand visual attention behavior in the context of driving. Some works used eye movement, head position changes, and facial features to predict the direction of visual attention. For example, Valenti et al. [8] enhanced the accuracy of gaze estimation using eye location and head pose. Fridman et al. [9] showed that facial landmarks inferring relative head movement improved visual attention estimation. These studies predicted a driver’s attention direction under task-specific considerations; however, they paid limited attention to the interaction between attention locations and the visual appearance of the environments.

As opposed to these works, Itti and Koch [4] addressed the advantages of image-based visual attention modeling, where scene understanding and object recognition strongly constrain the selection of attention locations. Visual attention modeling refers to a mechanism that selectively highlights the regions which contain the most important and conspicuous regions of visual scenes, also known as (bottom-up) saliency modeling. Existing saliency models have successfully captured visual attention in natural scenes. However, they usually show poor performance in driving situations that have complex dynamics [2,10]. There exist efforts to understand a driver’s visual attention associating with visual features. Pugeault and Bowden [2] demonstrated that the driver’s focus of attention strongly correlated with the regions related to a driver’s actions.

**Figure 1:**
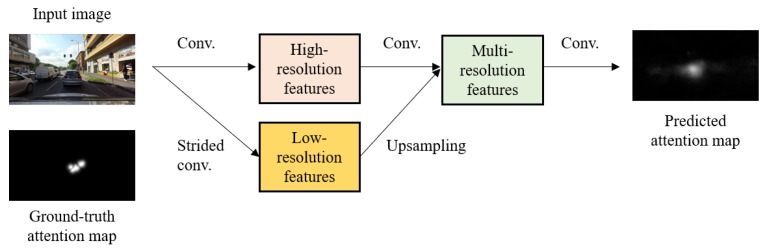
Where is a salient location required by a driver? Given images, this work aims to predict a driver’s attention location. The proposed framework transforms input images into their spatially corresponding attention maps. The output attention map where the map value at every pixel location directly represents a driver’s visual attention, abstracts from fused features of multiple scales.

Recently, the studies of predicting a driver’s attention locations have shown dramatic progress, owing to the development of deep convolutional neural network frameworks [10,11,12,13]. Existing attention prediction approaches based on deep neural networks leverage semantic segmentation networks that predict a class label among a set of predefined class labels for each pixel of an image. Transferring semantic segmentation to a driver’s attention prediction, the output of the attention prediction network is a spatial map where each pixel value represents a driver’s visual attention, as shown in Figure 1.

**Figure 2:**
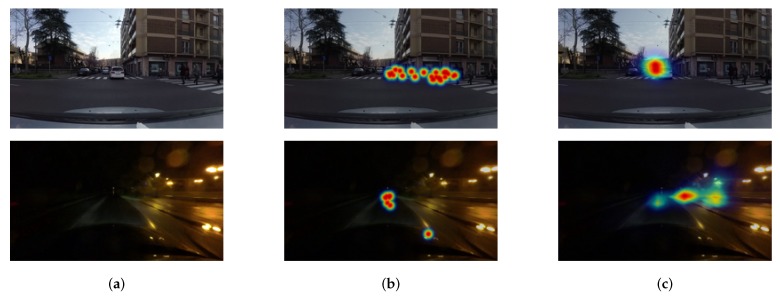
Example of failure case. (**a**) Input image. (**b**) Ground-truth attention map. (**c**) Predicted attention map. The predicted map failed to detect pedestrians on the crosswalk and traffic signs and localize the detected attention location.

Although existing deep attention prediction models produce decent results, one challenging issue of predicting attention locations is the reduced spatial resolution of feature maps. Typical semantic segmentation networks intrinsically shrink resolutions of feature maps using pooling or strided convolution throughout forward processes. While these operations increase the size of the receptive field (which helps to recognize visual information over large regions), they potentially lead to the loss of fine-grained features and the degradation of localization performance (see Figure 2). As the downsampling operations produce feature maps at reduced resolutions, dense prediction maps at the full image resolution should be reconstructed by reversing downsampling operations (e.g., upsampling, deconvolution, or fractionally strided convolution). However, the reconstruction procedure usually fails to recover already-lost-features, eventually causing prediction accuracy and localization degradation.

To overcome this coarse prediction problem, in this work, we adapt and extend a deep-high-resolution architecture that can learn and extract multi-scale features at the different spatial resolutions. The network generates not only local/low-level features at the full image resolution but also more global/high-level features at the full image resolution. It is achieved by extracting feature maps at multiple resolutions, by fusing the feature maps at the end of each stage, and by having multiple stages. Dense attention maps are finally predicted based on fused-multi-resolution feature maps at the full image resolution. We train the network using a widely used driver attention dataset [11] under the following considerations: First, unlike semantic segmentation networks, we approach attention estimation task as a regression problem by predicting continuous value (attentiveness) at each pixel. Regression-based approach is effective as it can determine penalty based on the distance between current prediction and ground-truth during training. Second, as visual attention datasets are strongly biased towards centers of images, we diversify attention locations by randomly cropping and augmenting training images. Augmenting data is useful in shifting attention locations to various positions of images, which is helpful to avoid overfitting to the center-biased solution.

The remainder of this paper is organized as follows. In Section 2, we review the related works regarding a driver’s attention prediction networks and semantic segmentation networks. In Section 3, we provide observations of visual attention behavior and develop a deep neural network framework based on the observations. Experimental setups and results are shown in Section 4, and concluding remarks are made in Section 5.

## 2. Related Works

### 2.1. Saliency Map Estimation and Visual Attention Prediction in Driving Scene

Many researchers have studied saliency map estimation methods to predict the importance of each region in given images [14,15]. Itti et al. proposed a framework to select a small number of interesting image locations for rapid scene analysis [16]. They determine these regions based on intensity contrast, color contrast, and local orientation contrast. Harel et al. proposed the graph-based visual saliency (GBVS) estimation method [17]. Given classic features (orientation, contrast, luminance), they compute activation maps and normalize the maps using the Markovian algorithm.

A driver’s attention location was traditionally estimated from the head/eye positions and angles [8,18]. Lethaus et al. showed that driver gaze information is useful to predict lane changing maneuvers, where they obtained gaze data using a head-mounted eye-tracking system (SMI iView X^TM^ HED) [18]. Valenti et al. proposed a method that integrates head pose tracker and eye locator to improve visual gaze estimation [8].

The work in [2] showed that a method which uses only pre-attentive vision, can predict driver behaviors such as braking and steering in real time. They also explored the relationship between driver attentive vision and random forest activation maps where the random forest model is learned to predict road context and driver action.

Recently, driver attention prediction studies have shown promising results by using deep neural networks. The work in [10,19] proposed a Bayesian framework that uses the fully convolutional neural networks (FCN) [20,21]. Palazzi et al. estimated attention maps using a multi-branch network that incorporates features from RGB images, segmentation maps, and optical flow [11]. They demonstrated that the accuracy of attention maps is improved by using motion information and semantic cues.

As critical driving moments are so rare, Xia et al. proposed the Berkeley DeepDrive Attention (BDD-A) dataset which contains selected videos based on braking events [12]. They proposed a network that composes of the AlexNet [22] and a convolutional long short-term memory (LSTM) that was trained by using a human weighted sampling (HWS) strategy to identify crucial frames. The work in [23] explored a way to interpret image regions that possibly influence vehicle control.

### 2.2. Convolutional Neural Networks for Pixel-Wise Prediction

Visual attention prediction can be accomplished by estimating the pixel-wise score of being conspicuous. As semantic segmentation has been addressed by predicting the probability of being each class for each pixel by many researchers [20,21,24,25,26,27], visual attention prediction has been approached by adapting the convolutional neural networks for semantic segmentation [10]. For pixel-wise prediction, Long et al. proposed FCN for semantic segmentation by replacing fully connected layers with convolutional layers in the neural networks for image classification [20,21].

As pooling or strided convolution in typical convolutional neural networks reduces the resolution of feature maps, it has been actively studied that recovering the representations to original resolution or maintaining the representations at original resolution [24,25,28]. In FCN framework, Long et al. increased the resolution of features maps using either bilinear interpolation (last layer) or learnable deconvolution filters (intermediate layers). Noh et al. [27] proposed the deconvolution network that consists of deconvolution and unpooling layers to recover the resolution of feature representations to that of an input image. U-Net [26] introduced additional links that couple layers of a contracting path and an expansive path. The contracting path is to extract feature maps by convolutions and max-pooling. The expansive path is to fuse low-level features and high-level features by concatenating upsampled feature maps from the previous layer and corresponding feature maps from the contracting path. Yu and Koltun [24] investigated the structural differences of semantic segmentation from image classification when repurposing to dense prediction. Designing a network specifically for dense prediction, they presented the context module based on dilated convolutions that support the expansion of the receptive field without losing resolution. The resolutions of feature maps were maintained by removing pooling and striding layers and by replacing all subsequent convolution layers with dilated convolution layers. The upsampling network (decoder) of SegNet [25] was designed for improving boundary localization while converting low-resolution feature maps to the outputs at the input resolution. Similar to the work in [27], SegNet memorized and reused max-pooling indices from the corresponding encoder feature map to precisely delineate boundaries. The final dense outputs were produced by convolving trained filters with feature maps upsampled using max-pooling indices. DeepLab [29,30] also addressed the issue of feature map resolution reduction. To keep resolution at the input resolution, the work replaced convolutional layers of VGG-16 network [31] by atrous convolution that has zeros between non-zeros filter tap except for the last pooling or convolutional layer. Similarly, the stride of the last pooling and convolutional layer set to 1 to avoid downsampling.

Along with high-resolution representation, multi-scale deep architectures have been also actively explored. Prior studies have shown that combining multi-scale features improves segmentation performance because incorporating global features with local features helps to understand scenes better. Zhao et al. [32] exploited the capability of global context information by aggregating multi-scale features. They empirically showed that fusing information from different sub-regions could invoke more powerful feature representation. Based on the observations of failure cases, the work proposed the pyramid scene parsing network (PSPNet) that contains the pyramid pooling module that is composed of multiple pooling units, followed by upsampling and concatenation layers to form the final feature representation. DeepLab [29,30] also improved segmentation performance by fusing features at different level representations. The full-resolution feature maps are recovered by a combination of atrous spatial pyramid pooling (ASPP) [29] and bilinear interpolation to refine segmentation results.

RefineNet [33] proposed the multi-path network that exploited various levels of features at different stages of convolution and recursively combined them to obtain a high-resolution prediction up to a quarter of original image size. To produce outputs of original image size, the final dense map was upsampled using bilinear interpolation. Pohlen et al. [34] proposed the network that has the residual stream at full image resolution and the pooling stream at low-resolution. The two processing streams were coupled using full-resolution residual units (FRRUs) that contain pooling, convolution, and upsampling. The image cascade network ICNet [35] integrated multi-resolution feature maps with the cascade label guidance strategy. The network took cascade image inputs at different resolutions (i.e., low-, medium and high resolution) and adopts cascade feature fusion units that combine two different resolution feature maps. DenseASPP network developed for complementing scale variation in street scenes, which requires a larger receptive field and features at various levels. The network densely connected atrous convolutional layers in a cascade fashion, where the output of each atrous convolution layer feeds to all atrous convolution layers ahead. By modifying the connection of atrous convolution layers, the network could have a denser feature pyramid and a larger receptive field compared with the original ASPP [29].

Inspired by these studies, we present a deep neural network framework for driver visual attention prediction. The differences in our work from other studies are: first, unlike existing semantic segmentation networks, our proposed network is designed specifically for visual attention prediction in the context of driving. As scenes perceived by drivers are extremely wide and complex to understand, some regions posing certain visual challenges such as objects in various size are tremendously more difficult to be recognized as attention locations. Moreover, data bias of on-road visual attention datasets further hinders accurate attention location prediction. Based on these facts, we believe that existing semantic segmentation and saliency detection models are limited to the study of visual attention detection in driving situations. Therefore, a better understanding of the limitations of transferring state-of-the-art algorithms is essential. To this end, we investigate key factors that should be considered in producing better prediction accuracy for on-road datasets and further provide empirical observations on the key factors. The observations reveal that fusing multiple level features at the full image resolution improves prediction accuracy and localization performance. However, existing models for driver visual attention prediction mostly capture features at the reduced resolutions of the original input image and yield prediction accuracy degradation. Therefore, we develop a deep neural network framework that maintains low- and high-level features at high resolution and aggregates these high-to-low features through multiple stages. Our experimental results demonstrate that high-resolution features indeed improve performance over state-of-the-art methods.

## 3. Proposed Approach

### 3.1. Key Considerations

To accurately predict a driver’s visual attention in more realistic and complex situations, we consider three factors that significantly impact prediction accuracy. The three factors are described below.

Multi-Scale Features: In computer vision, low- and high-resolution representations of a visual field interpret as the proxies of central and peripheral visions (see Section 1). Features captured from low-resolution represent coarse visual information over a large area and provide context-level scene understanding. Features captured from high resolution contain fine details over a small area that allows detecting precise pixel-wise interest regions. It is well accepted that combining features at multiple scales would improve the network ability to predict correct labels by capturing both local and global contexts.

Typical attention prediction networks effectively capture deep, coarse, and contextual features using successive pooling operations or strided convolutions. However, the spatial dimensions of output feature maps are significantly reduced as the number of pooling or strided convolution layers increases, causing that fine and pixel-wise visual information is potentially lost. This loss of fine-grained features leads to mislabeling small attentive regions as inattentive regions and delineating inaccurate boundaries of attentive regions. Furthermore, to predict the saliency of each pixel on an input image, the full-resolution outputs should be reproduced from the low-resolution feature maps. This dense output reproduction is usually carried out by upsampling, deconvolution, or fractionally strided convolution until all downsampling effects are eliminated. However, the reproducing procedure often fails to recover visual features that are already lost during feature extraction steps. Therefore, capturing features at high resolution and fusing them with features at low-resolution is necessary to improve prediction performance. Indeed, given a specific scene on the top row of Figure 2, objects of large size or high contrast are predicted as attention locations, whereas small or remote objects are predicted as inattention locations. Moreover, although attention locations are correctly detected, the detected region is localized inaccurately on the bottom row of Figure 2. In addition, as shown in the examples of Figure 2, scenes perceived by drivers cover a wide range of regions, and the scales objects largely vary in the scene. Consequently, extracting features at multiple scales is more important to represent scene dynamics of on-road datasets.

*Discrete vs. Continuous Outputs*: A cost function is a measure of how well networks predict outputs for a given problem. The choice of a cost function is an important aspect of designing a deep neural network model. Specifically, cost functions are used to compute the loss of prediction and update hyperparameters to reduce the loss on the next evaluation. A training procedure searches optimal solutions based on the loss, thereby the performance of the network is significantly dependent on the cost function.

By applying semantic segmentation networks to a driver’s attention prediction, the problem is formulated as a multi-class classification, which labels are discrete intensity values of images. The goal of training networks for classification is to maximize margins between classes. To maximize class separation, a training procedure usually uses (SoftMax) cross-entropy loss in practice, which highly penalizes misclassification errors. However, a human driver perceives seamless and continuous scenes, not discrete levels. Owing to this property of human visual perception, the strategy has been employed to smooth sampled attention points and generate dense saliency maps [11,36]. The blurred representation of visual attention indicates that attentive locations are distributed over pixels with small intensity differences. Moreover, when training networks using SoftMax cross-entropy loss, a training procedure magnifies only one class that gives the highest probability of data being in the class. This maximization of class separation ignores class distances and treats all negative classes equally. This is undesirable for the continuous scene assumption where the distance between current output and ground-truth determines prediction performance. To this end, positing an attention prediction task as a regression problem by predicting continuous values is more suitable in the context of driving rather than a classification problem.

**Figure 3:**
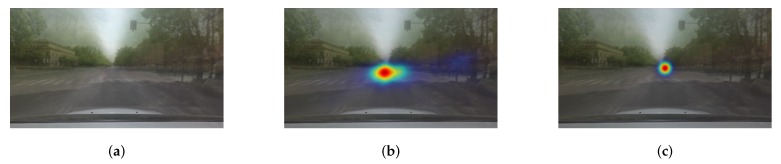
Center-biased attention property of on-road dataset. (**a**) Mean frame. (**b**) Mean attention map. (**c**) Predicted attention map by Gaussian blob. The mean attention map of (**b**) shows that a driver’s attention tends to direct the center of the scene, although it contains objects possibly attracts, for example, traffic signals and signs. The Pearson correlation coefficient of naive estimation between Gaussian blob in (**c**) and ground-truth is 0.5477, which reflects the center-biased data. Please note that the mean frame and attention map are generated by taking the average over the entire frames and ground-truth attention maps of an example video.

*Biased Dataset*: It is well known that visual attention is biased toward the center of natural scenes, so that regions of interest tend to be placed near the center of images [37,38]. This center bias tendency is prominent in saliency datasets collected by vehicles on roads where vanishing points of roads are usually placed at the center of images. To be concrete, a dataset bias can be demonstrated simply by generating Gaussian blobs at the center of an image and comparing them against the ground-truth attention maps. The naive Gaussian center model indeed yields acceptable results, as experimentally verified in Figure 3.

The experiment demonstrates that a training procedure for attention location detector easily emphasizes visual features found in the center regions, because of the high bias in on-road visual attention datasets. The overfitting to hit-the-center solution indicates that prediction performance significantly relies on the network’s ability to selectively allocating attention to particular regions. Therefore, training networks to avoid learning visual features from trivial center solutions is essential.

### 3.2. Model Description

As described in Section 3.1, there are potential issues with applying typical neural network models to on-road visual attention prediction. To demonstrate the observations and being motivated by work [28], we employ and train the fully convolutional network to be specialized at predicting driver attention. The proposed network fuses multi-scale features and defines nontrivial local-to-global and detail-to-context representations across layers, resulting in more precise spatial prediction.

**Figure 4:**
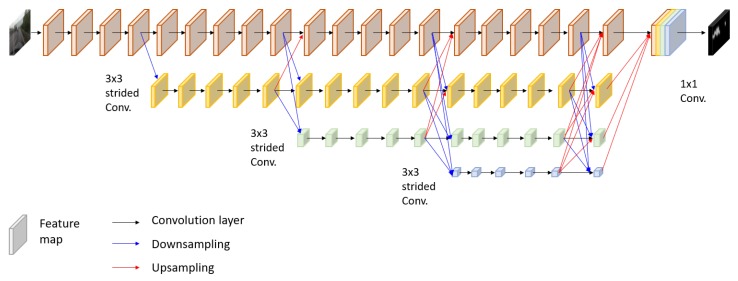
Network architecture. There are four sequential sub-networks that represent features at different resolutions. Black, blue, and red arrows denote convolution layer, downsampling layer, and upsampling layer, respectively.

The overall architecture of the developed network is depicted in Figure 4. The entire network composes of four sub-networks that extract salient visual features from maps at full image resolution and maps at lower resolutions. The uppermost sub-network maintains the resolution of an input image to the final output, not suffering from downsampling that potentially causes fine feature loss. In parallel to the first sub-network, three sub-networks generate feature maps at the decreased resolutions by 1/2, 1/4, and 1/8 using strided 3×3 convolutions. To produce the final dense output, feature maps at different resolutions are merged by upsampling and convolution. Specifically, low-resolution feature maps increase the resolution up to the feature map size of upper sub-networks by bilinear upsampling then convolve with the high-resolution output every four convolutions. At each stage, features captured at multiple resolutions are fused, and the fused features are propagated to the next stage. The structure is effective in producing more accurate results by repeatedly combining multi-scale features and keeping the original resolution. The final prediction maps are generated by progressively fusing such multi-scale features over all sub-networks in a fully convolutional manner.

To train the network, we draw on regression principles using L2-loss, as described in Section 3.1. Let $t={\left[\;y_{1},\cdots ,y_{M}\;\right]}^{T}$ be the vectorized version of *M* pixels from a ground-truth attention map and $o={\left[\;{\hat{y}}_{1},\cdots ,{\hat{y}}_{M}\;\right]}^{T}$ be the corresponding probability of attention estimated. Then the loss function L is defined, as follows:

(1) $$\begin{array}{c} L\left(o_{n},\;t_{n}\;\right)=\frac{1}{N}\sum_{n=1}^{N}||\;o_{n}-t_{n}\;{\left|\right|}_{2}^{2}\;, \end{array}$$

where $\left|\right|\cdot {\left|\right|}_{2}$ is the Euclidean norm, and the subscript *n* denotes the *n*-th vector of t and o in a batch of *N* inputs. The loss is averaged over the batch to erode extreme values and to effectively control a learning rate. In practice, we followed the training strategy described by in [28]. The proposed network pre-trained for ImageNet classification problem [39] and fine-tuned using the stochastic gradient descent (SGD) optimization with the initial learning rate of 0.01. The momentum and weight decay set to 0.9 and 0.0005, respectively. The learning was ended at about 11 K iterations, and the batch size *N* in (1) set to 4 on 4 GPUs due to the GPU memory constraints. The hard hyperbolic tangent function was chosen as the activation function of the last fully connected layer. The analysis of the effects of different activation functions is addressed in Section 4.

One way to handle the center-biased estimation problem (See Section 3.1) is shifting attention regions in various locations of the scenes. Thus, we randomly cropped the original input images and ground-truth attention maps to prevent the network from learning center-biased estimation. The input images from 1920×1080 to 960×540 using random scaling in the range of $\left[0.5,2\right]$ and applying horizontal flipping.

## 4. Experimental Setup and Results

### 4.1. Dataset

We validate our model using the DR(eye)VE dataset provided in [11]. The dataset consists of 5-minute-long 74 video sequences captured by 8 drivers using a roof-mounted camera, *Garmin VirbX*. We chose the dataset because it is suitable to test a wide variety of driving situations, including the scenes of landscapes, weather conditions, and daytime. In the dataset, the attention maps were captured by using a wearable eye-tracking device, *SMI ETG 2w* Glasses. The attention locations were selected as the maximum values of the projected gaze points over a temporal sliding window. For training and testing, we followed the data separation scheme used in [11]. We split into the first 37 sequences for training and the last 37 sequences for testing. In the experiments, we excluded the frames marked as errors [11] to thoroughly study prediction performance without any secondary effects. For training, we also excluded the frames that are collected when the speed of the vehicle was zero.

### 4.2. Results

The performance of our model is compared against deep neural network-based models, traditional saliency detection algorithms, and a baseline. We compare with the state-of-the-art deep neural network-based models reported using the dataset in Section 4.1. The models are the network in [10] (“Tawari”), the network in [12] (“HWS”), and the network in [11] (“Multi-branch”). As discussed in Section 3.1, a naive attention estimation at the center of an image could yield better inference than other saliency models. For this reason, we also consider “baseline” attention prediction by averaging ground-truth attention maps over all training sequences. In addition to the deep models and the baseline, we compare performance with the traditional well-known saliency detection algorithms: the model in [16] (“Itti”) and graph-based visual saliency (“GBVS”) [17].

To assess prediction performance, we use a metric known as the Pearson correlation coefficient [40,41]. The correlation coefficient (CC) for a test image is computed as follows:

(2) $$\begin{array}{c} CC=\frac{{\left(t^{\prime }-\bar{t^{\prime }}\right)}^{T}\cdot \left(o^{\prime }-\bar{o^{\prime }}\right)}{\sqrt{||\;t^{\prime }-\bar{t^{\prime }}{\;||}_{2}^{2}\cdot ||\;o^{\prime }-\bar{o^{\prime }}\;{\left|\right|}_{2}^{2}}}\;\;, \end{array}$$

where

(3) $$\begin{array}{c} t^{\prime }={\left[\;t_{1}^{\prime },\cdots ,t_{M}^{\prime }\;\right]}^{T}={\left[\frac{t_{1}-m_{t}}{\sigma_{t}},\cdots ,\frac{t_{M}-m_{t}}{\sigma_{t}}\right]}^{T},\;\;\bar{t^{\prime }}=\left[\;m_{t^{\prime }},\cdots ,m_{t^{\prime }}\;\right],\; \end{array}$$

(4) $$\begin{array}{c} o^{\prime }={\left[o_{1}^{\prime },\cdots ,o_{M}^{\prime }\right]}^{T}={\left[\frac{o_{1}-m_{o}}{\sigma_{o}},\cdots ,\frac{o_{M}-m_{o}}{\sigma_{o}}\right]}^{T},\;\;\bar{o^{\prime }}=\left[\;o_{t^{\prime }},\cdots ,o_{t^{\prime }}\;\right]. \end{array}$$

the vectors $t^{\prime }$ and $o^{\prime }$ are the normalized versions of t (ground-truth attention) and o (estimated attention), respectively; *m* and σ denote the mean and standard deviation of either a ground-truth attention map or an estimated attention map (e.g., $m_{t}$ is the mean of ground-truth and $m_{\hat{o}}$ is the mean of estimation). The vectors ${\bar{t}}^{\prime }$ and ${\bar{o}}^{\prime }$ are the vectors which contain all equal elements, where $m_{t}^{\prime }$ and $m_{o}^{\prime }$ denote the average values of the elements of $t^{\prime }$ and $o^{\prime }$. The metric CC is suitable for our assumption that driver attention locations can be expressed as a distribution over image locations (We did not consider other common measurements NSS and AUC as they are computed based on discrete values [42]).

**Figure 5:**
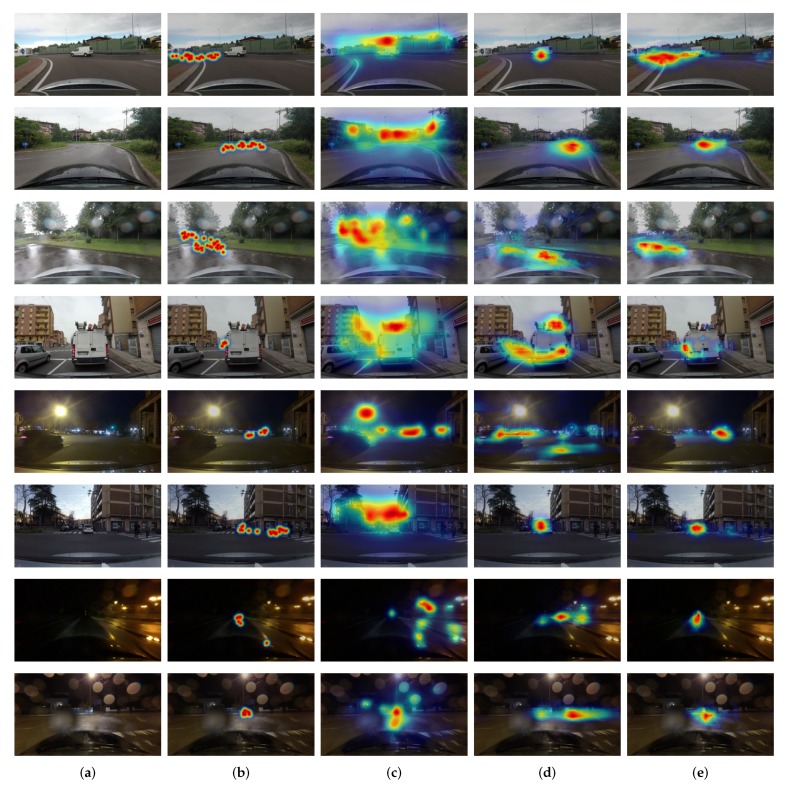
Visual comparisons of the predicted attention maps. The output attention maps overlay with the input images and scale to RGB components. (**a**) Input Image. (**b**) Ground-truth attention map. (**c**) Attention map by GBVS [17]. (**d**) Attention map of state of the art [11]. (**e**) Attention map of the proposed model. The proposed model can detect contextual and pixel-wise labels consistently and accurately.

We summarize the quantitative results of attention prediction in Table 1. The reported scores are the average of CC values in (2) over all test images. The proposed model improves prediction performance of about 7.14% relative to the state-of-the-art model. The traditional models have very weak correlations with driver attention (CC≤0.2). This is somehow expected because the traditional models estimate attentive regions only based on discernible visual features. The baseline performs better than the traditional models, but it appears to be mostly due to the center-biased frames, as demonstrated in Figure 3. The deep neural network-based models predict drivers’ attention better and show higher scores. Among them, the proposed model predicts more accurate attentive locations, as proven in the visual comparisons of Figure 5. In general, the proposed model shows good localization performance. Furthermore, the proposed model can detect small regions, for example, pedestrians and a traffic signal in the images of the fifth and sixth rows in Figure 5 and predicted well for non-center-biased frames in the examples of first to third rows. This demonstrated that the model successfully detects contextual and pixel-wise attention maps by fusing low-level and high-level features repeatedly. As shown in the examples of the last two images, the model can robustly predict attention location even in poor light and weather conditions.

**Figure 6:**
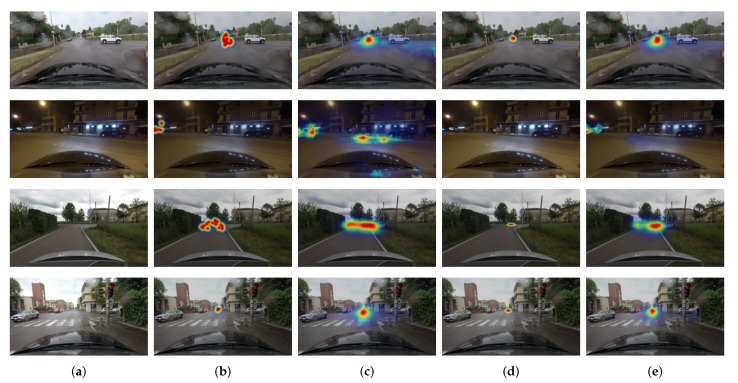
Visual comparisons of the predicted attention maps generated by different activation functions. (**a**) Input Image. (**b**) Ground-truth attention map. (**c**) Linear activation function. (**d**) Hyperbolic tangent. (**e**) Hard hyperbolic tangent.

We compared the performance of the proposed model with three activation functions of the last layer. These functions are linear, hyperbolic tangent, and hard hyperbolic tangent. The quantitative comparison results are tabulated in Table 2 in terms of the average CC values over all test images. The proposed model improved prediction performance over the state-of-the-art method for all different activation functions. The average CC score of hyperbolic tangent function is the lowest, while the scores of linear and hard hyperbolic tangents are comparable. The score of hard hyperbolic tangent implies that bounding the outputs to a certain range is profitable to improve performance. The visual comparisons are also shown with different activation functions in Figure 6. The examples in Figure 6 indicate that hard hyperbolic tangent function result in better localization performance than linear function due to its finite dynamic range.

Additionally, we found that the CC score of highway scenes is relatively higher than those of downtown and countryside scenes regardless of different activation functions, as shown in Table 3. This fact can be explained as human drivers are more distracted by irrelevant information in a complex environment. This also interpreted as drivers are more cautious and less distracted while driving at high speed. The result is consistent with driver visual behavior that drivers tend to constantly stare at the road and follow road features such as lane markings. The proposed model using hard hyperbolic tangent function well captured the driver’s visual behavior. The driver’s visual behavior is well captured by the proposed model using the hard hyperbolic tangent function. The hard hyperbolic tangent function is more likely to produce high scores in the narrow regions where drivers focus more at high speed, as it shows good localization performance.

**Figure 7:**
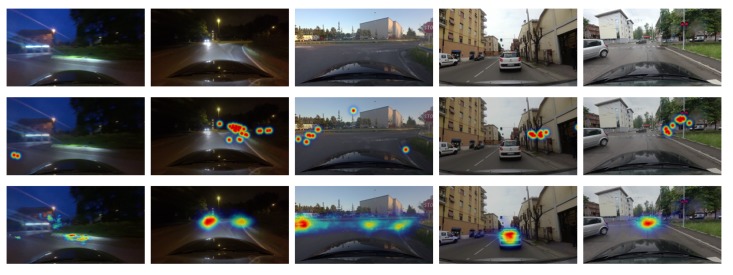
Failure cases. From top to bottom: input images, ground-truth attention map, and estimated attention map by the proposed model.

We also show some of the failure cases in Figure 7. The attention locations in the first and second cases are obviously challenging to detect due to extreme lighting sources, which are appeared to be the most distinct features in the images. The third case shows a situation where the driver gazed at multiple locations. Although the proposed network predicts the most attentive location, it fails to detect less attentive locations. In the fourth and fifth cases, the network predicts the car in front as the attention location, but the driver looked at objects (e.g., trees, traffic signal) on the sidewalk. Because human drivers are likely to pay more attention to cars in front than other objects on the sides, the network disagrees. Based on these observations, we believe that further investigation between visual dynamics of multiple attention locations [11,12] and object important ranking [1] may lead to additional insights for more faithful attention prediction.

## 5. Conclusions

As the human visual system plays a critical role in making proper decisions for safe driving, understanding driver visual attention from images could be a crucial key to assist intelligent vehicle systems where a self-driving car is required to move safely. Thus, in this paper, we investigated human driver visual attention and developed a deep convolutional neural network framework to improve driver attention prediction. First, we showed that fusing high- and low-level features at the full image resolution enhanced localization performance and spatial prediction accuracy. Moreover, further investigation for on-road driving datasets demonstrated that the regression approach and data augmentation to avoid data bias yielded better prediction accuracy. Lastly, we experimentally verified that the proposed model improves prediction accuracy compared to the standard and the state-of-the-art driver prediction attention models. 

## Figures and Tables

**Table 1:** Performance of the proposed model against other models in terms of CC in (2). Please note that the score of HWS model was measured from the network that trained using the selected 200 ten-second long video clips from the DR(eye)VE dataset [11]. The model was fine-tuned with their own dataset proposed in [12] and tested for the DR(eye)VE dataset. Except for the model, other networks were trained and tested using the DR(eye)VE dataset.

Model	Baseline	Itti [16]	GBVS [17]	Tawari [10]	HWS [12]	Multi-Branch [11]	Proposed
Average CC	0.47	0.16	0.20	0.55	0.51	0.56	0.60

**Table 2:** Analysis of attention performance with different activation functions.

Activation Function	Linear	Hyperbolic Tangent	Hard Hyperbolic Tangent
Average CC	0.59	0.57	0.60

**Table 3:** Analysis of attention performance in different scenes and different activations. The average speeds are 30.77 km/h, 50.40 km/h, and 78.76 km/h for downtown, countryside, and highway scenes, respectively.

Activation	Linear	Hyperbolic Tangent	Hard hyperbolic Tangent
Scene			
Downtown	0.55	0.53	0.54
Countryside	0.57	0.57	0.55
Highway	0.62	0.61	0.65

## Abbreviations

The following abbreviations are used in this manuscript:

ADAS	Advanced Driver-Assistance System
GBVS	Graph-Base Visual Saliency
FCN	Fully Convolutional Network
BDD-A	Berkeley DeepDrive Attention
LSTM	Long Short-Term Memory
RefineNet	Multi-Path Refinement Network
ASPP	Atrous Spatial Pyramid Pooling
PSPNet	Pyramid Scene Parsing Network
FRRU	Full-Resolution Residual Units
ICNet	Image Cascade Network
SGD	Stochastic Gradient Descent
GPU	Graphics Processing Unit
NSS	Normalized Scanpath Saliency
AUC	Area under ROC Curve
